# Epilepsy alters brain networks in patients with insular glioma

**DOI:** 10.1111/cns.14805

**Published:** 2024-06-17

**Authors:** Qifeng He, Zuocheng Yang, BoWen Xue, Xinyu Song, Chuanhao Zhang, ChuanDong Yin, Zhenye Li, Zhenghai Deng, Shengjun Sun, Hui Qiao, Jian Xie, Zonggang Hou

**Affiliations:** ^1^ Department of Neurosurgery, Beijing Tiantan Hospital Capital Medical University Beijing China; ^2^ Department of Neuroradiology, Beijing Neurosurgical Institute, Beijing Tiantan Hospital Capital Medical University Beijing China; ^3^ Department of Radiology, Beijing Tiantan Hospital Capital Medical University Beijing China; ^4^ Department of Neurophysiology, Beijing Neurosurgical Institute Capital Medical University Beijing China

**Keywords:** brain networks, epilepsy, glioma, insula glioma

## Abstract

**Aims:**

We intend to elucidate the alterations of cerebral networks in patients with insular glioma‐related epilepsy (GRE) based on resting‐state functional magnetic resonance images.

**Methods:**

We collected 62 insular glioma patients, who were subsequently categorized into glioma‐related epilepsy (GRE) and glioma with no epilepsy (GnE) groups, and recruited 16 healthy individuals matched to the patient's age and gender to form the healthy control (HC) group. Graph theoretical analysis was applied to reveal differences in sensorimotor, default mode, visual, and executive networks among different subgroups.

**Results:**

No significant alterations in functional connectivity were found in either hemisphere insular glioma. Using graph theoretical analysis, differences were found in visual, sensorimotor, and default mode networks (*p* < 0.05). When the glioma located in the left hemisphere, the degree centrality was reduced in the GE group compared to the GnE group. When the glioma located in the right insula, the degree centrality, nodal efficiency, nodal local efficiency, and nodal clustering coefficient of the GE group were lower than those of the GnE group.

**Conclusion:**

The impact of insular glioma itself and GRE on the brain network is widespread. The networks altered by insular GRE differ depending on the hemisphere location. GRE reduces the nodal properties of brain networks than that in insular glioma.

## Graphical abstract

The networks altered by insular glioma‐related epilepsy (GRE) differ depending on the hemisphere location. GRE reduces the nodal properties of brain networks than that in insular glioma.

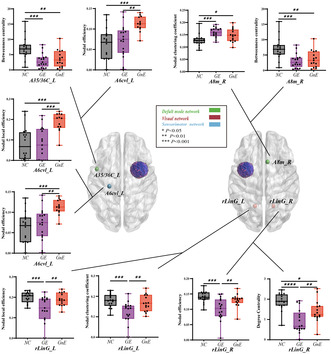

## 1 INTRODUCTION

Glioma‐related epilepsy (GRE) is a common manifestation accompanying glioma, occurring in approximately 60% of glioma patients.^1^ Superficial tumor location and isocitrate dehydrogenase (IDH) mutations are frequently implicated factors in the development of GRE.^2^ Both epilepsy and glioma induce changes in functional networks. Previous studies have reported associations between temporal and frontal GRE and alterations in functional connectivity.^3^ As an integral part of the cortical structure, the insula plays a role in various functions, including cognition and sensorimotor activities.^4^ Similar to other lobes GRE, insular GRE may disrupt the relationships between the insula and other brain regions. Consequently, there is a significant interest in investigating insular glioma and insular GRE. However, our understanding of the altered functional connectivity in insula glioma and insular GRE remains limited.

Resting‐state MRI blood oxygen level‐dependent (BOLD) signal measures changes in the brain's blood oxygen content, enabling the calculation of functional connections between different brain regions.^5^ Graphs are data structures composed of nodes and edges linking them.^6^ In a graphical representation of a brain network, each node represents a specific brain region, while edges signify the functional interactions between two brain regions. In recent years, there has been a growing interest in employing graph metrics to characterize aberrant large‐scale brain networks.^7^, ^8^ Previous studies have indicated that patients with insular epilepsy show an increase in the strength of cerebral network connections and a decrease in path length.^9^ However, the combined impact of glioma and epilepsy on cerebral networks is more intricate. There are fewer studies related to insular GRE. It remains uncertain whether insular glioma and insular GRE influence cerebral networks in a similar manner. Therefore, relying only on previous studies may impede antiepileptic therapy in patients with newly diagnosed glioma. Therefore, it is crucial to conduct further analysis to better understand the effects of insular glioma itself and insular GRE on brain networks, providing essential insights for the preoperative management of seizure occurrences. To address this issue more effectively, the present study retrospectively enrolled both healthy individuals and patients with glioma to investigate the changes in brain networks associated with insular GRE and glioma.

## 2 METHODS

The study has been evaluated and approved by the ethics community of Beijing Tiantan Hospital, Capital Medical University (KY 2020–146‐02).

### 2.1 Participants

This study conducted a retrospective analysis of patients diagnosed with insular glioma at the Department of Neurosurgery, Beijing Tiantan Hospital, between July 2019 and June 2022. Patient information, including age, gender, histopathology, IDH mutation status, and seizure history, was extracted from hospital records. Inclusion criteria were as follows: (1) age over 18 years; (2) right‐handed; (3) tumor primarily located in the insula; (4) patients with documented epilepsy; (5) histopathological confirmation of glioma; and (6) the absence of a history of brain disease. Exclusion criteria included: (1) antiepileptic drug usage exceeding 30 days; (2) head movement exceeding 3 mm; (3) the presence of multifocal glioma; and (4) uncertain history of epilepsy.

### 2.2 MRI acquisition

All MRI images were acquired from a 3 T MR scanner (MAGNETOM Prisma, Siemens). The acquisition parameters are as follows: (1) T2 fluid‐attenuated inversion recovery (FLAIR): echo time (TE) 581 ms; repetition time (TR) 5000 ms; flip angle (FA) 120°; Field of View (FOV) 192 × 256 mm; voxel size 1 × 1 × 1 mm; (2) rs‐fMRI: TE 35/30 ms; TR 750/2000 ms; FOV 220 × 220/192 × 192 mm; FA, 80°/90°; voxel size, 2.4 × 2.4 × 2.4/3.0 × 3.0 × 5.0 mm.

### 2.3 Image preprocessing progress

Lesion areas were manually delineated on T2 FLARE images by a neurosurgeon with 20 years of experience and produced as tumor masks using ITK‐SNAP.^10^ All tumor masks were subsequently normalized to the Montreal Neurological Institute (MNI) template using FSL.^11^ Rs‐fMRI data was preprocessed using GRETNA.^12^ Major steps include: (1) the first five volumes data were removed; (2) slice timing; (3) realignment to correct head movement; (4) functional images were normalized to EPI template;^13^ (5) images were smoothed with a Gaussian kernel of 4 mm full‐width at half maximum; (6) linear temporal detrending; (7) regression of nuisance variables (white matter signal: with WMMask3mm; CSF signal: with CSFMask3mm; head motion: Friston—24 parameters);^14^ and (8) temporal filtering (0.01–0.08 Hz).

### 2.4 Functional connectivity and the graph theory

The brain of each subject was partitioned into 210 cortical regions and 36 subcortical regions based on the human Brainnetome Atlas.^15^ Based on previous studies, four subnetworks related to insula function were extracted to compute functional connectivity: the visual, left/right sensorimotor, left/right executive, and left/right default mode networks.^16^ To mitigate the impact of glioma, the contralateral network was selected for calculating the sensorimotor, executive, and default mode networks. Subnetwork details are provided in Tables S1–S7.

We calculated the Pearson's correlation coefficient for the average time series of each region, subsequently creating the functional connectivity matrix. To enhance the statistical properties, we applied Fisher's *Z* transform to all functional connectivity matrices. These matrices were then transformed into binary undirected graphs, spanning a range of sparsity values (0.16 to 0.4, with intervals of 0.01). The minimum sparsity threshold is estimated using the Gretna_get_rmax command in GRETNA.^17^ The area under the curve (AUC) is highly sensitive to the brain disease's topology. We calculated the AUC for the topological features within the defined threshold range. Global and node topological features of the functional networks are calculated using the GRETNA^12^ package(Network Analysis toolbox): (1) global properties: global efficiency, local efficiency; (2) small‐world networks: the clustering coefficient (CP), Gamma, Lambda, Sigma and the shortest length (LP); and (3) nodal properties: the nodal efficiency, nodal local efficiency, nodal clustering coefficient, degree centrality, and betweenness centrality.

### 2.5 Statistical analyses

Various statistical methods were employed to assess intergroup differences in demographic and clinical characteristics. Depending on the nature of data, two‐sample *t*‐tests, one‐way analysis of variance (ANOVA), and chi‐square tests were used to compare characteristics. Student's *t*‐test was applied to compare functional connectivity differences between GE and GnE groups, and the results were subsequently post hoc test using the false discovery rate (FDR). The AUC of global and nodal topological features was compared by one‐way ANOVA (corrected by false‐positive‐adjustment).^18^ If the corrected results show a difference, the least significant difference (LSD) test for post hoc pairwise global and node properties comparisons. *p* < 0.05 was considered significantly different.

## 3 RESULTS

**FIGURE 1:**
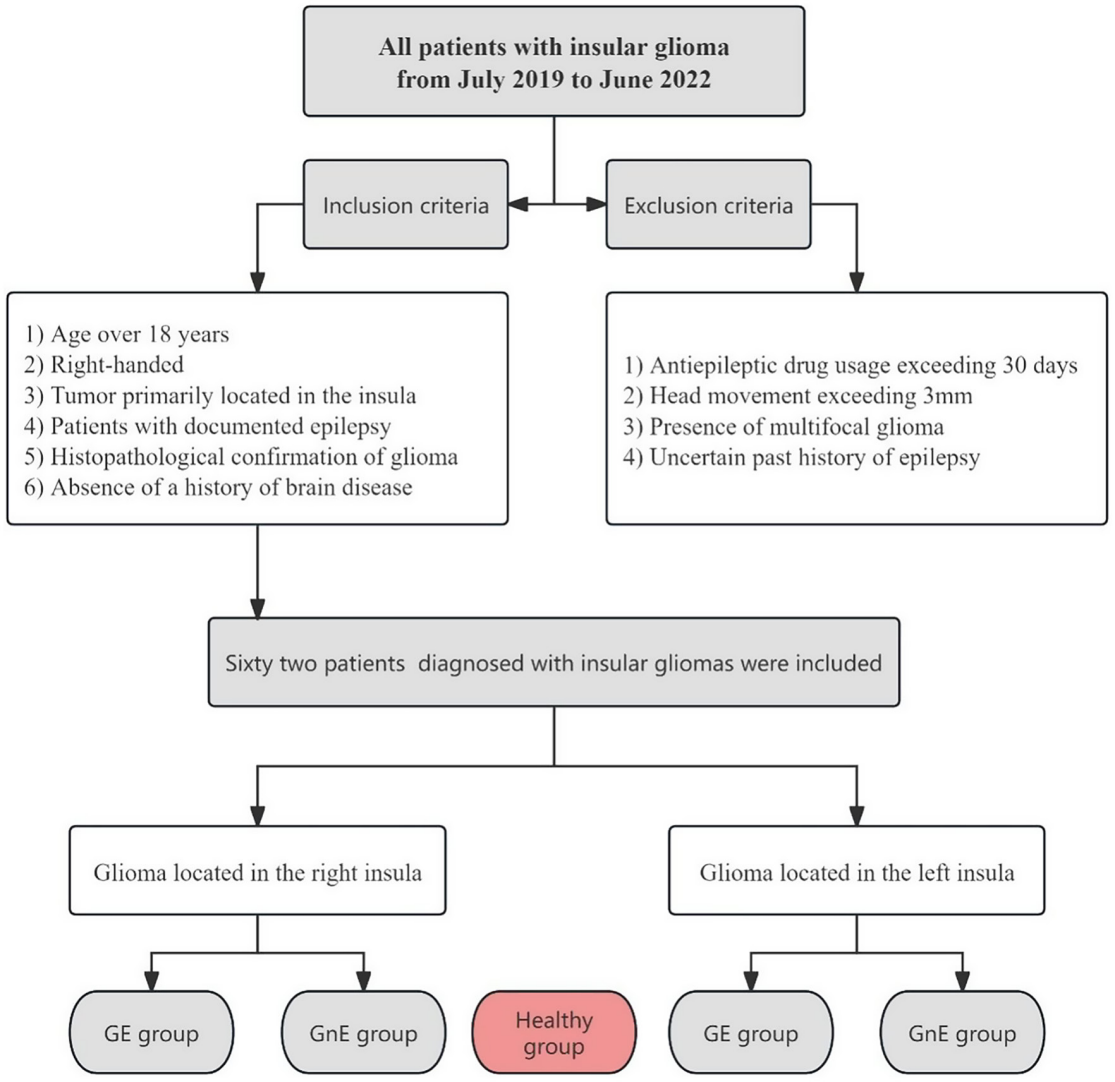
Pipeline of enrolling patients.

**FIGURE 2:**
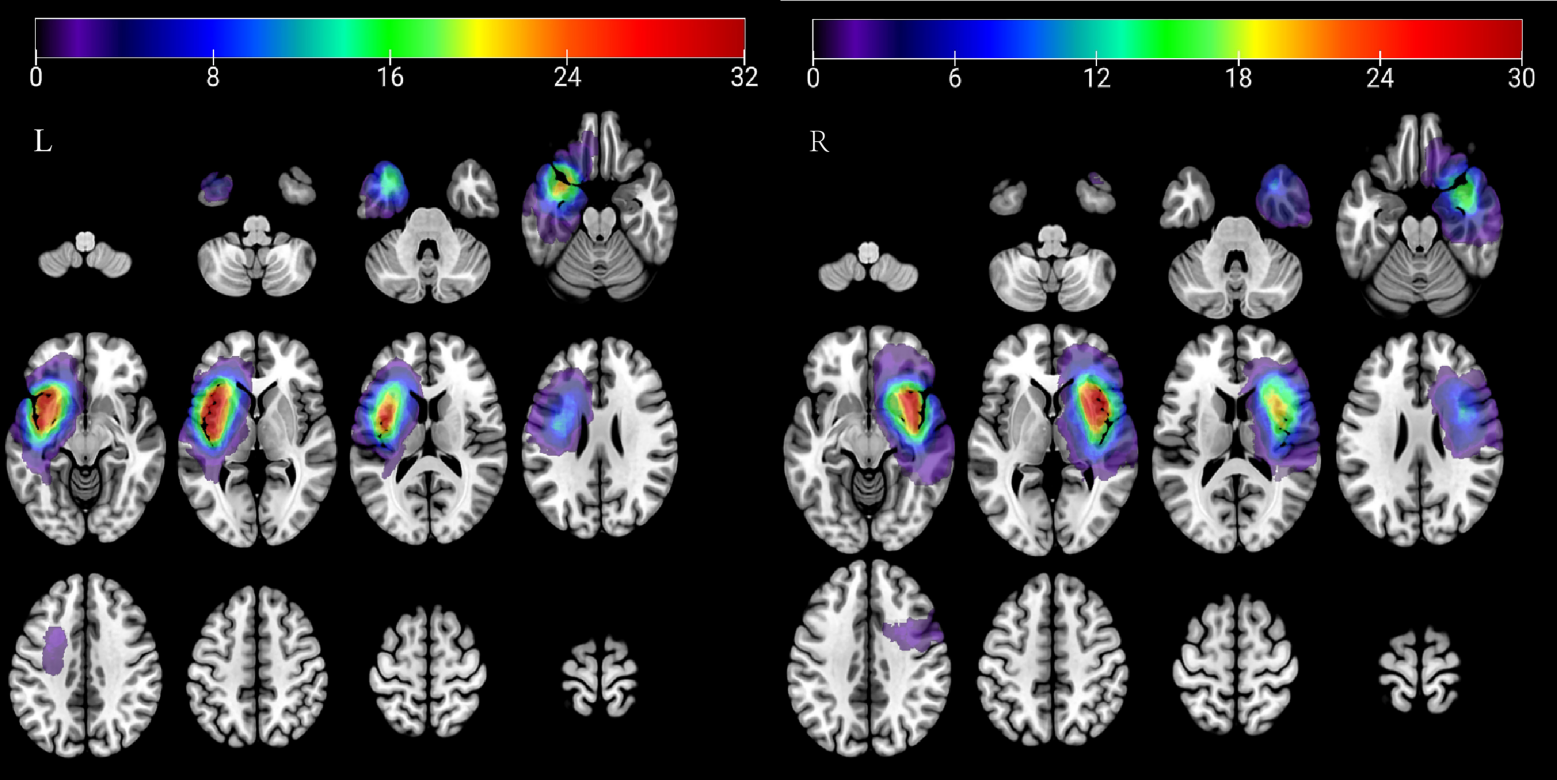
Lesion overlap area for (A) left insular glioma and (B) right insular glioma.

Sixty‐two patients (*n* = 30, R; *n* = 32, L) diagnosed with insular glioma were included in the study and statistically compared with 16 HC, while 21 patients were excluded (Figure 1). Thirty‐four patients were included in the GE group based on the presence or absence of epilepsy (17 patients with insular glioma in the right hemisphere and 17 in the left hemisphere), and 28 patients were divided into the GnE group (13 patients with insular glioma in the right hemisphere and 15 in the left hemisphere). The overlapping areas of lesions in both right and left glioma located in the insula (Figure 2). No significant differences were observed among the three groups concerning age, gender, and handedness. Additionally, there were no disparities in IDH mutation status, tumor volume, and pathology between the GE and GnE groups. (Tables 1 and 2).

**TABLE 1:** Demographic and clinical characteristics of patients with right insular gliomas.

Demographic characteristics	Patients		HC	*p*‐values
	GE	GnE		
Number	17	13	16	NA
Age, years^a^	43.94 ± 2.41	40.77 ± 3.65	42.2 ± 2.9	0.753
Gender, F/M	5/12	4/9	6/10	0.872
Handedness, R/L, *n*	17/0	13/0	16/0	NA
Tumor volume, cm^3^ ^a^	53.95 ± 11.08	43.26 ± 14.14	NA	0.551
Tumor grade				
Grade 2	9	9	NA	—
Grade 3	6	5		
Grade 4	2	3		
IDH status, Mutation/Wild‐type	12/5	10/3	NA	>0.999

*Note*: The *p*‐values were determined by a one‐way ANOVA for age, a *χ* ^2^ test for gender, handedness, histopathology, IDH status, and a *t*‐test for tumor volume.

^a^ Data are means ± standard errors.

**TABLE 2:** Demographic and clinical characteristics of patients with left insular gliomas.

Demographic characteristics	Patients		HC	*p*‐values
	GE	GnE		
Number	17	15	16	NA
Age, years^a^	45.2 ± 3.1	46.1 ± 3.3	42.2 ± 2.9	0.654
Gender, F/M	6/11	6/9	6/10	0.96
Handedness, R/L, *n*	17/0	15/0	16/0	NA
Tumor volume, cm^3^ ^a^	62.33 ± 10.63	40.74 ± 8.88	NA	0.135
Tumor grade				
Grade 2	8	10	NA	—
Grade 3	5	4		
Grade 4	4	1		
IDH status, Mutation/Wild‐type	11/6	12/3	NA	0.444

*Note*: The *p*‐values were determined by a one‐way ANOVA for age, a *χ* ^2^ test for gender, handedness, histopathology, IDH status, and a *t*‐test for tumor volume.

^a^ Data are means ± standard errors.

### 3.1 Functional connectivity

The study revealed no significant difference in functional connectivity between the GE and GnE groups of the four sub‐networks in right and left sided glioma after FDR correction.

### 3.2 Global topological properties

**FIGURE 3:**
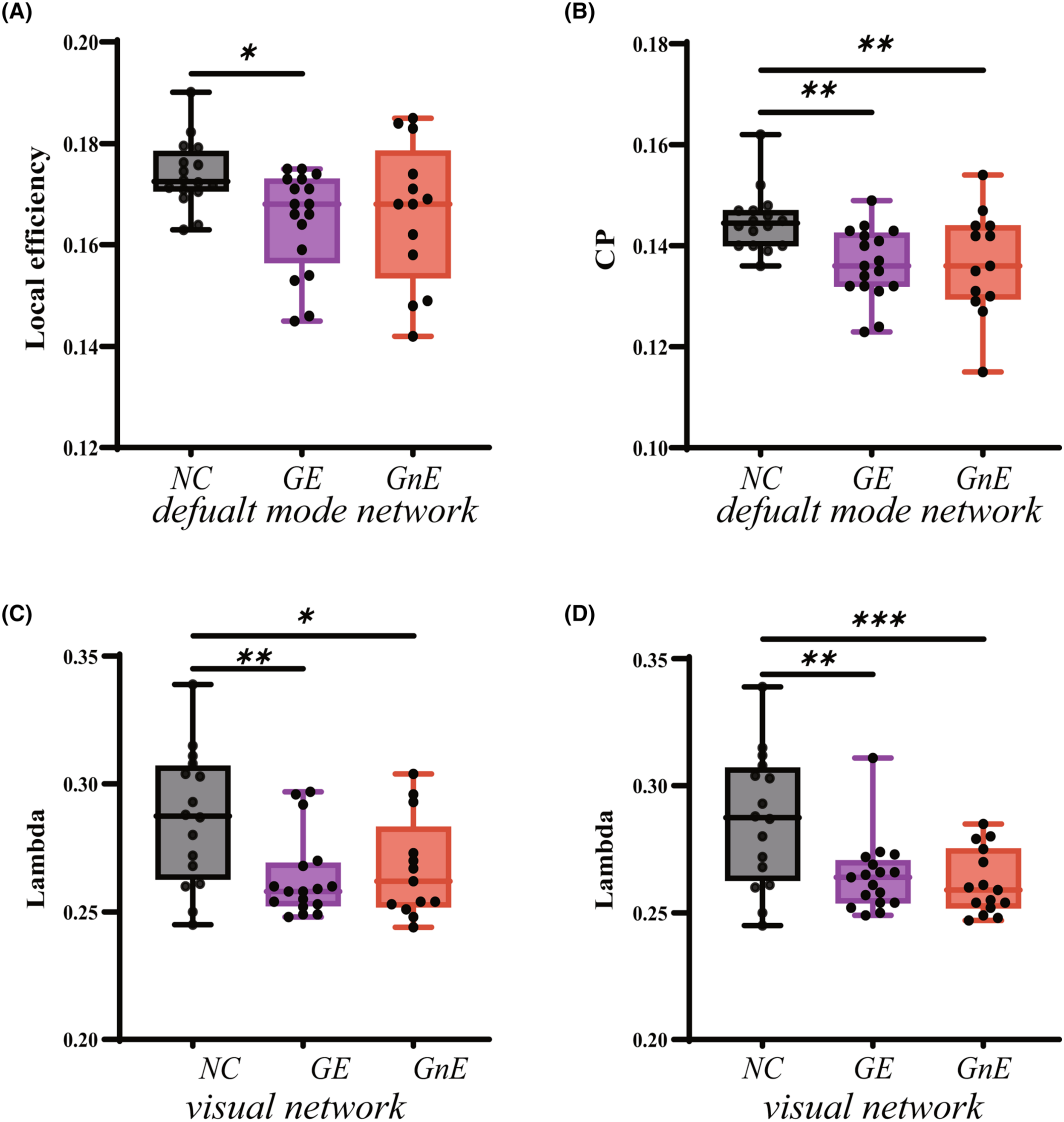
Results of global topology property changes among the three groups (*p* < 0.05, corrected). (A) Local efficiency of default mode network when glioma located in the right insula. (B) CP of default mode network when glioma located in the right insula. (C) Lambda of visual network when glioma located in the right insula. (D) Lambda of visual network when glioma located in the left insula. The GE group = glioma‐related epilepsy group. The GnE group = glioma with no related epilepsy. The HC group = healthy control group. * *p* < 0.05, ** *p* < 0.01, *** *p* < 0.001 (LSD corrected)

In cases where the glioma situated in the right insula, the three groups exhibited variations in the local efficiency of the default mode network (*p* = 0.0355) as determined by one‐way ANOVA (Figure 3A, Table S8). After a post‐hoc correction with LSD correction, the local efficiency of GE (0.165 ± 0.002) was significantly lower compared to that of the HC group (0.174 ± 0.002, *p* = 0.0143). When glioma located in the left insular lobe, none of the four networks detected global properties differences.

### 3.3 Small‐world properties

Regarding small‐world properties, both the CP (*p* = 0.0049) of the default mode network (Figure 3B, Table S8) and the lambda (*p* = 0.0069) of the visual networks (Figure 3C, Table S9) exhibited differences among the three groups. After a post hoc correction with LSD correction, the lambda of the visual network in the GE (0.263 ± 0.004, *p* = 0.0030) and GnE (0.267 ± 0.007, *p* = 0.0163) groups was significantly lower than that in the HC (0.287 ± 0.007) group. Furthermore, the CP values of the default mode networks in the GE (0.136 ± 0.002, *p* = 0.0031) and GnE groups (0.137 ± 0.003, *p* = 0.007) were also significantly lower than those in the HC group (0.145 ± 0.002).

When glioma located in the left insula, differences in the visual networks’ lambda (*p* = 0.0009) were observed among the three groups, similar to the findings in the right insula (Figure 3D, Table S10). After a post hoc correction with LSD correction, the Lambda of the visual network in the GE (0.264 ± 0.004, *p* = 0.0015) and GnE (0.262 ± 0.003, *p* = 0.0007) groups was significantly lower than that in the HC group (0.287 ± 0.007).

The global properties of the remaining networks (left/right sensorimotor and left/right executive network) did not differ among the three groups (*p* < 0.05, corrected).

### 3.4 Nodal topological properties

**FIGURE 4:**
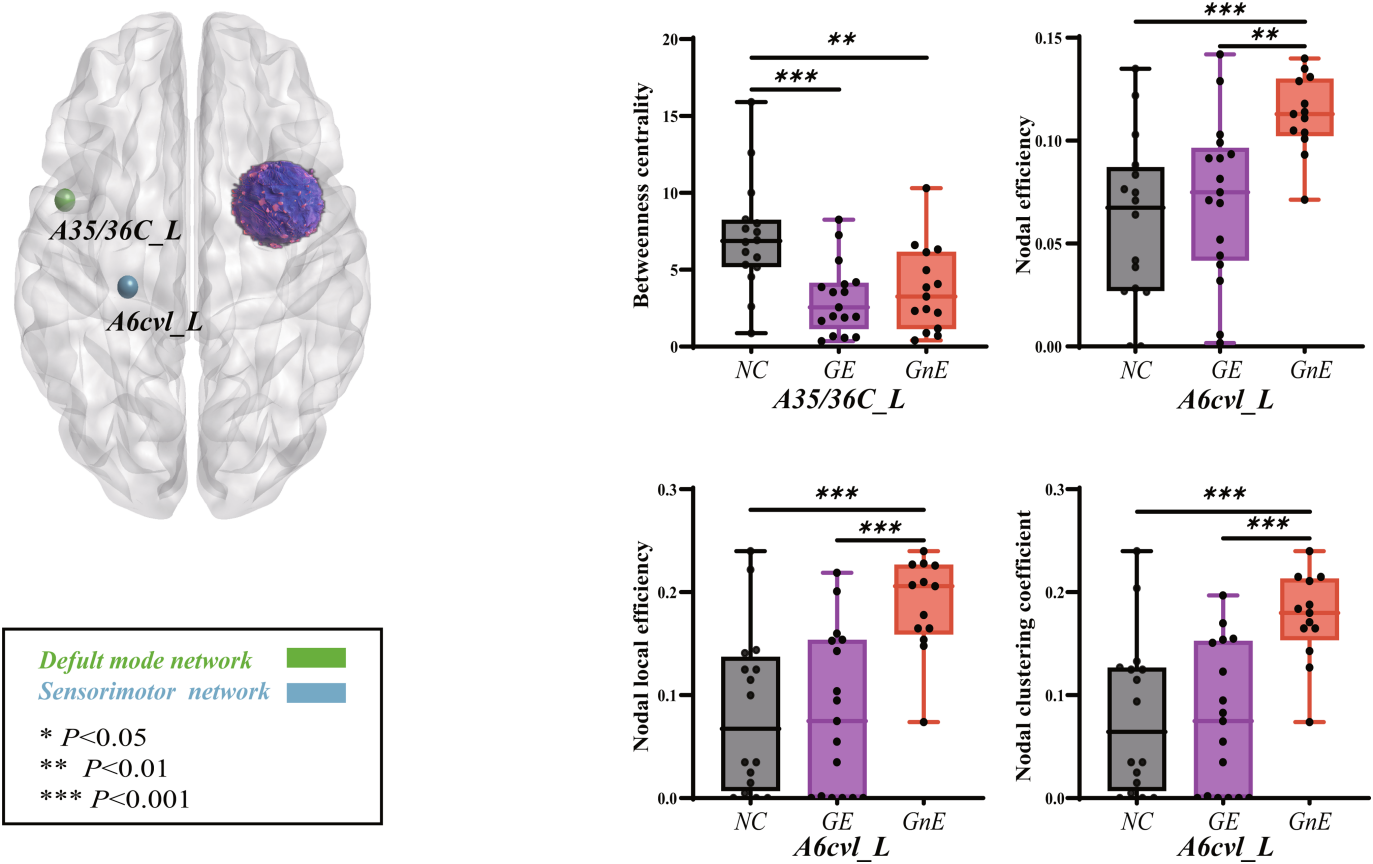
Results of nodal topology property changes among the three groups when glioma is located in the right hemisphere (*p* < 0.05, corrected). The GE group = glioma‐related epilepsy group. The GnE group = glioma with no related epilepsy. The HC group = healthy control group. * *p* < 0.05, ** *p* < 0.01, *** *p* < 0.001 (LSD corrected).

Nodal properties of the sensorimotor and the default mode networks differed when the glioma in the right insular lobe (Figure 4, Tables S11–S14). After correcting for false‐positive tests, A6cvl_L (caudal ventrolateral area 6) showed differences in the sensorimotor network. Nodal efficiency (*p* = 0.0008), nodal local efficiency (*p* = 0.0003), and nodal clustering coefficient (*p* = 0.0003) varied among the three groups. In A6cvl_L, GnE exhibited higher nodal efficiency (0.113 ± 0.005) than GE (0.072 ± 0.009, *p* = 0.0029) and HC groups (0.061 ± 0.010, *p* = 0.0003). Nodal local efficiency in the GnE group (0.187 ± 0.013) was significantly higher than GE (0.082 ± 0.019, *p* = 0.0002) and HC groups (0.083 ± 0.020, *p* = 0.0003). Additionally, nodal clustering coefficient in the GnE group (0.175 ± 0.012) was higher than GE (0.076 ± 0.017, *p* = 0.0002) and HC groups (0.080 ± 0.019, *p* = 0.0004). In the default mode network, betweenness centrality of A35/36c_L (caudal area 35/36, *p* = 0.0012) differed after correction for false‐positive tests. In A35/36c_L, the GE (0.906 ± 0.303) had weaker betweenness centrality than the GnE (4.361 ± 1.206, *p* = 0.0104) and the HC groups (5.593 ± 1.070, *p* = 0.0004).

**FIGURE 5:**
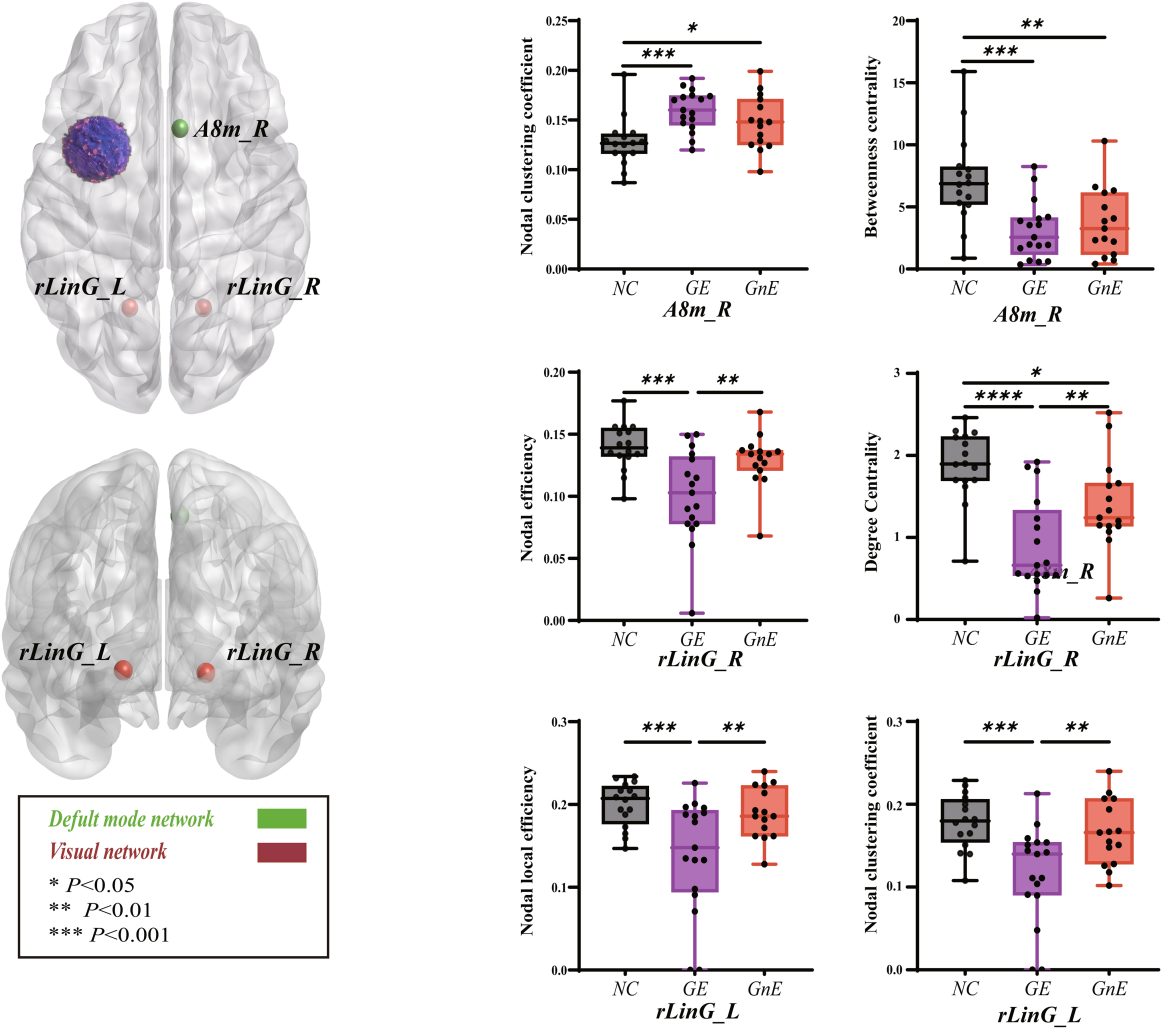
Results of nodal topology property changes among the three groups when glioma is located in the left hemisphere (*p* < 0.05, corrected). The GE group = glioma‐related epilepsy group. The GnE group = glioma with no related epilepsy. The HC group = healthy control group. * *p* < 0.05, ** *p* < 0.01, *** *p* < 0.001 (LSD corrected).

When the glioma in the left insular lobe, nodal properties of the visual and default mode networks differed (Figure 5, Tables S15–S20). After correcting for false‐positive tests, rLinG_L(R) (rostral lingual gyrus) showed differences in degree centrality (*p* < 0.0001), nodal efficiency (*p* = 0.0004), nodal local efficiency (*p* = 0.0012), and nodal clustering coefficient (*p* = 0.0007). In rLinG_L, GE (0.893 ± 0.139) had lower degree centrality than the GnE (1.397 ± 0.144, *p* = 0.0095) and HC group (1.882 ± 0.108, *p* < 0.0001). The nodal efficiency of the GE group (0.101 ± 0.009) was also lower than the GnE (0.129 ± 0.006, *p* = 0.0052) and the HC groups (0.140 ± 0.005, *p* = 0.0002). Regarding rLinG_R, the nodal local efficiency of the GE (0.140 ± 0.017) was lower than the GnE (0.190 ± 0.008, *p* = 0.0040) and the HC groups (0.200 ± 0.007, *p* = 0.0006). Nodal clustering coefficient of the GE (0.117 ± 0.014) was also lower than the GnE (0.166 ± 0.010, *p* = 0.0037) and the HC groups (0.178 ± 0.008, *p* = 0.0003). A8m_R (Medial area 8) in the default mode network has differences in nodal clustering coefficient (*p* = 0.0013) and betweenness centrality (*p* = 0.0005) after correction for false‐positive tests. In A8m_R, the nodal clustering coefficient was lower in the HC group (0.127 ± 0.006) than that in the GE (0.160 ± 0.005, *p* = 0.0003) and the GnE groups (0.148 ± 0.007, *p* = 0.0225). On the contrary, betweenness centrality was significantly higher in the HC group (7.133 ± 0.899) than that in the GE (3.093 ± 0.561, *p* = 0.0003) and the GnE groups (3.712 ± 0.713, *p* = 0.0021).

After correction, nodal properties of the remaining network (left/right executive network) were not different between the three groups (*p* < 0.05, corrected).

## 4 DISCUSSION

This study explores the distinctions in functional networks between insular glioma and insular GRE. Our study identified alterations in the sensorimotor, default mode, and visual networks among patients with insular glioma and GRE. The specific impact varied based on the hemisphere of injury: right insular glioma and GRE predominantly affected the sensorimotor and default mode networks, whereas left insular glioma and GRE primarily altered the visual and default mode networks. Compared to the nodal properties of insular glioma itself, GRE reduces the nodal attributes of the brain network.

Differential alterations observed in the three sub‐networks illustrate the impact of insular glioma and GRE on brain networks. The function of the insula involves sensorimotor and cognitive functions but is not limited to them.^4^, ^19^ Recent studies have found that the insula shows higher activation responses in visual and auditory modalities.^20^, ^21^ Our study identified that insular glioma exhibit a capacity to reorganize visual and sensorimotor networks, consistent with findings from prior research.^22^, ^23^ Alterations in the topological properties of the visual network were predominantly observed in the left brain, while changes in the topological properties of the sensorimotor network were evident in the right brain. This discrepancy may be attributed to functional lateralization, a fundamental principle of brain organization.^24^, ^25^ The triple network model, consisting of the executive network, default mode network, and salience network, holds significant sway over cognitive processes. The anterior insula governs the activity of the default mode network.^26^, ^27^ Our results also indicate that insular glioma and GRE can induce changes in the default mode network. The salience network, anchored in the anterior insula, assumes a pivotal role in initiating network switching.^28^, ^29^, ^30^ Tumor‐induced disruptions of cortico‐cortical connections may disrupt this process.^31^ Therefore, we posited that insular glioma might indirectly impact the default mode network through the saliency network.

The strength of functional connections between brain regions, often referred to as functional connectivity, is a measure of the degree of synchrony.^32^ In contrast to the observed differences in functional connectivity between glioma and GRE in the temporal and frontal lobes, no significant distinctions were identified in the context of insular glioma. Small‐world properties gauge the degree of integration and segregation in brain functions.^33^, ^34^ Both the GE and GnE groups exhibited smaller small‐world properties in visual and default mode networks than the HC group. Visual and default mode networks undergo reorganization in the presence of glioma, affirming the critical role of the insula in these networks.

The nodal properties highlight the differences between the GE and GnE groups. When the glioma located on the right side, the nodal properties of the GE group were significantly lower than those of the GnE group, but no significant difference was observed compared to the HC group. This finding suggests that the glioma activated nodes, while GRE inhibited them. When the glioma was on the left side, the nodal properties of the GE group were also lower than those of the GnE group. However, the nodal properties of the GnE group were comparable to those of healthy individuals. In summary, GRE inhibits node activation.

However, a major limitation of the study is that glioma induces changes in the structure and integrity of the peritumoral vasculature, consequently influencing the vascular microenvironment.^35^, ^36^ In this study, computations were conducted utilizing contralateral brain networks for three networks, excluding the visual network. As a result, it was challenging to discern network alterations on the glioma‐affected side. In future investigations, we intend to address this limitation and explore the specific alterations within the glioma‐affected networks. Another limitation of our study lies in the challenge of assessing the impact of antiepileptic drugs on the functional network. The influence of antiepileptic drugs on functional networks is known to be dose‐dependent, capable of either activation or inhibition.^37^, ^38^ In our investigation, patients with epilepsy were administered antiepileptic drugs, but the duration of usage was relatively short. In addition, due to the limited sample size, we did not categorize the epilepsies to explore the differences between the different types of epilepsy. Future studies will continue to include more patients to discuss how different epilepsy types alter the insula glioma brain network.

## 5 CONCLUSIONS

Our study indicates that the impact of insular glioma itself and GRE on the brain network is widespread. Insular glioma and GRE in distinct hemispheres induce modifications in various functional networks. In addition, GRE reduced brain network nodal properties than that in glioma. These findings contribute significantly to our comprehension of the alterations in functional networks associated with glioma and GRE. Moreover, the study offers novel insights into strategies for both preoperative and postoperative epilepsy prevention in individuals with insular glioma. In our future research, we will explore the combination of molecular mechanisms and imaging to deeply analyze the link between glioma and epilepsy. We hope that by elucidating the specific mechanisms by which glioma triggers epilepsy, we can provide a scientific basis for the innovation and advancement of therapeutic approaches.

## AUTHOR CONTRIBUTIONS

The conceptual foundation of the research: JX and ZGH. Data acquisition and analysis: ZCY, BWX, XYS, CHZ, ZYL, CDY, HQ, and SJS. Data interpretation: QFH and ZGH. Figures and drafting creation: QFH. Writing the first draft: QFH and ZGH. Supervision research: ZHD and JX. All contributing authors have examined and endorsed the finalized manuscript and jointly agree to its submission for journal publication.

## FUNDING INFORMATION

This study was supported by the National Natural Science Foundation of China (82172028).

## CONFLICT OF INTEREST STATEMENT

The authors declared that they have no conflicts of interest.

## Supporting information


Table S1.

## Data Availability

The data that support the findings of this study are available from the corresponding author upon reasonable request.
