# Supplementary material for: Epilepsy alters brain networks in patients with insular glioma
Source: CNS Neurosci Ther. 2024 Jun 17;30(6):e14805. doi: 10.1111/cns.14805 (PMC11183176; doi:10.1111/cns.14805)
Supplement: Supplementary file 1 — Table S1. [file CNS-30-e14805-s001.docx]

**Supplemental Tables**

**Table S1. Nodes and their Montreal Neurological Institute (MNI) locations in the left sensorimotor network.**

| **Area** | **Anatomical and modified Cyto-architectonic descriptions** | **Left hemisphere**  X, Y, Z |
| --- | --- | --- |
| *A6m_L* | *medial area 6* | -6, -5, 58 |
| *A4hf_L* | *area 4(head and face region)* | -49, -8, 39 |
| *A6cdl_L* | *caudal dorsolateral area 6* | -32, -9, 58 |
| *A4ul_L* | *area 4(upper limb region)* | -26, -25, 63 |
| *A4t_L* | *area 4(trunk region)* | -13, -20, 73 |
| *A4tl_L* | *area 4(tongue and larynx region)* | -52, 0, 8 |
| *A6cvl_L* | *caudal ventrolateral area 6* | -49, 5, 30 |
| *A1/2/3ll_L* | *area1/2/3 (lower limb region)* | -8, -38, 58 |
| *A4ll_L* | *area 4, (lower limb region)* | -4, -23, 61 |
| *A1/2/3ulh_Lf* | *area 1/2/3(upper limb, head and face region)* | -50, -16, 43 |
| *A1/2/3tonIa_L* | *area 1/2/3(tongue and larynx region)* | -56, -14, 16 |
| *A2_L* | *area 2* | -46, -30, 50 |
| *A1/2/3t_Lru* | *area1/2/3(trunk region)* | -21, -35, 68 |
| *A23d_L* | *dorsal area 23* | -4, -39, 31 |
| *A23v_L* | *ventral area 23* | -8, -47, 10 |
| *A24cd_L* | *caudodorsal area 24* | -5, 7, 37 |
| *A23c_L* | *caudal area 23* | -7, -23, 41 |
| *A32sg_L* | *subgenual area 32* | -4, 39, -2 |
| *mPMtha_L* | *pre-motor thalamus* | -18, -13, 3 |
| *Stha_L* | *sensory thalamus* | -18, -23, 4 |

**Table S2. Nodes and their Montreal Neurological Institute (MNI) locations in the right sensorimotor network.**

| **Area** | **Anatomical and modified Cyto-architectonic descriptions** | **Right hemisphere**  X, Y, Z |
| --- | --- | --- |
| *A6m_R* | *medial area 6* | 7, -4, 60 |
| *A4hf_R* | *area 4(head and face region)* | 55, -2, 33 |
| *A6cdl_R* | *caudal dorsolateral area 6* | 33, -7, 57 |
| *A4ul_R* | *area 4(upper limb region)* | 34, -19, 59 |
| *A4t_R* | *area 4(trunk region)* | 15, -22, 71 |
| *A4tl_R* | *area 4(tongue and larynx region)* | 54, 4, 9 |
| *A6cvl_R* | *caudal ventrolateral area 6* | 51, 7, 30 |
| *A1/2/3ll_R* | *area1/2/3 (lower limb region)* | 10, -34, 54 |
| *A4ll_R* | *area 4, (lower limb region)* | 5, -21, 61 |
| *A1/2/3ulhf_R* | *area 1/2/3(upper limb, head and face region)* | 50, -14, 44 |
| *A1/2/3tonIa_R* | *area 1/2/3(tongue and larynx region)* | 56, -10, 15 |
| *A2_R* | *area 2* | 48, -24, 48 |
| *A1/2/3tru_R* | *area1/2/3(trunk region)* | 20, -33, 69 |
| *A23d_R* | *dorsal area 23* | 4, -37, 32 |
| *A23v_R* | *ventral area 23* | 9, -44, 11 |
| *A24cd_R* | *caudodorsal area 24* | 4, 6, 38 |
| *A23c_R* | *caudal area 23* | 6, -20, 40 |
| *A32sg_R* | *subgenual area 32* | 5, 41, 6 |
| *mPMtha_R* | *pre-motor thalamus* | 12, -14, 1 |
| *Stha_R* | *sensory thalamus* | 18, -22, 3 |

**Table S3. Nodes and their Montreal Neurological Institute (MNI) locations in the left default mode network.**

| **Area** | **Anatomical and modified Cyto-architectonic descriptions** | **Left hemisphere**  X, Y, Z |
| --- | --- | --- |
| *A8m_L* | *medial area 8* | -5 ,15, 54 |
| *A8dl_L* | *dorsolateral area 8* | -18, 24, 53 |
| *A9l_L* | *lateral area 9* | -11, 49, 40 |
| *A6dl_L* | *dorsolateral area 6* | -18, -1, 65 |
| *A6m_L* | *medial area 6* | -6, -5, 58 |
| *A9m_L* | *medial area 9* | -5, 36, 38 |
| *A10m_L* | *medial area 10* | -8, 56, 15 |
| *A9/46d_L* | *dorsal area 9/46* | -27, 43, 31 |
| *A9/46v_L* | *ventral area 9/46* | -41, 41, 16 |
| *A8vl_L* | *ventrolateral area 8* | -33, 23, 45 |
| *A6vl_L* | *ventrolateral area 6* | -32, 4, 55 |
| *A20iv_L* | *intermediate ventral area 20* | -45, -26, -27 |
| *A37elv_L* | *extreme lateroventral area37* | -51, -57, -15 |
| *A20r_L* | *rostral area 20* | -43, -2, -41 |
| *A20il_L* | *intermediate lateral area 20* | -56, -16, -28 |
| *A37vl_L* | *ventrolateral area 37* | -55, -60, -6 |
| *A20cl_L* | *caudolateral of area 20* | -59, -42, -16 |
| *A20cv_L* | *caudoventral of area 20* | -55, -31, -27 |
| *A35/36r_L* | *rostral area 35/36* | -27, -7, -34 |
| *A35/36c_L* | *caudal area 35/36* | -25, -25, -26 |
| *TL_L* | *area TL (lateral PPHC, posterior parahippocampal gyrus)* | -28, -32, -18 |
| *A28/34_L* | *area 28/34 (EC, entorhinal cortex)* | -19, -12, -30 |
| *TI_L* | *area TI (temporal agranular insular cortex)* | -23, 2, -32 |
| *TH_L* | *area TH (medial PPHC)* | -17, -39, -10 |
| *A5l_L* | *lateral area 5* | -33, -47, 50 |
| *A23d_L* | *dorsal area 23* | -4, -39, 31 |
| *A24rv_L* | *rostroventral area 24* | -3, 8, 25 |
| *A24cd_L* | *caudodorsal area 24* | -5, 7, 37 |
| *A23c_L* | *caudal area 23* | -7, -23, 41 |

**Table S4. Nodes and their Montreal Neurological Institute (MNI) locations in the right default mode network.**

| **Area** | **Anatomical and modified Cyto-architectonic descriptions** | **Right hemisphere**  X, Y, Z |
| --- | --- | --- |
| *A8m_R* | *medial area 8* | 7, 16, 54 |
| *A8dl_R* | *dorsolateral area 8* | 22, 26, 51 |
| *A9l_R* | *lateral area 9* | 13, 48, 40 |
| *A6dl_R* | *dorsolateral area 6* | 20, 4, 64 |
| *A6m_R* | *medial area 6* | 7, -4, 60 |
| *A9m_R* | *medial area 9* | 6, 38, 35 |
| *A10m_R* | *medial area 10* | 8, 58, 13 |
| *A9/46d_R* | *dorsal area 9/46* | 30, 37, 36 |
| *A9/46v_R* | *ventral area 9/46* | 42, 44, 14 |
| *A8vl_R* | *ventrolateral area 8* | 42, 27, 39 |
| *A6vl_R* | *ventrolateral area 6* | 34, 8, 54 |
| *A20iv_R* | *intermediate ventral area 20* | 46, -14, -33 |
| *A37elv_R* | *extreme lateroventral area37* | 53, -52, -18 |
| *A20r_R* | *rostral area 20* | 40, 0, -43 |
| *A20il_R* | *intermediate lateral area 20* | 55, -11, -32 |
| *A37vl_R* | *ventrolateral area 37* | 54, -57, -8 |
| *A20cl_R* | *caudolateral of area 20* | 61, -40, -17 |
| *A20cv_R* | *caudoventral of area 20* | 54, -31, -26 |
| *A35/36r_R* | *rostral area 35/36* | 28, -8, -33 |
| *A35/36c_R* | *caudal area 35/36* | 26, -23, -27 |
| *TL_R* | *area TL (lateral PPHC, posterior parahippocampal gyrus)* | 30, -30, -18 |
| *A28/34_R* | *area 28/34 (EC, entorhinal cortex)* | 19, -10, -30 |
| *TI_R* | *area TI (temporal agranular insular cortex)* | 22, 1, -36 |
| *TH_R* | *area TH (medial PPHC)* | 19, -36, -11 |
| *A5l_R* | *lateral area 5* | 35, -42, 54 |
| *A23d_R* | *dorsal area 23* | 4, -37, 32 |
| *A24rv_R* | *rostroventral area 24* | 5, 22, 12 |
| *A24cd_R* | *caudodorsal area 24* | 4, 6, 38 |
| *A23c_R* | *caudal area 23* | 6, -20, 40 |

**Table S5. Nodes and their Montreal Neurological Institute (MNI) locations in the left executive network.**

| **Area** | **Anatomical and modified Cyto-architectonic descriptions** | **Left hemisphere**  X, Y, Z |
| --- | --- | --- |
| *A8dl_L* | *dorsolateral area 8* | -18, 24, 53 |
| *A9m_L* | *medial area 9* | -5, 36, 38 |
| *A9/46d_L* | *dorsal area 9/46* | -27, 43, 31 |
| *IFJ_L* | *inferior frontal junction* | -42, 13, 36 |
| *A46_L* | *area 46* | -28, 56, 12 |
| *A9/46v_L* | *ventral area 9/46* | -41, 41, 16 |
| *A8vl_L* | *ventrolateral area 8* | -33, 23, 45 |
| *A6vl_L* | *ventrolateral area 6* | -32, 4, 55 |
| *A10l_L* | *lateral area10* | -26, 60, -6 |
| *IFS_L* | *inferior frontal sulcus* | -47, 32, 14 |
| *A45r_L* | *rostral area 45* | -49, 36, -3 |
| *A12/47l_L* | *lateral area 12/47* | -41, 32, -9 |
| *A21c_L* | *caudal area 21* | -65, -30, -12 |
| *A37vl_L* | *ventrolateral area 37* | -55, -60, -6 |
| *A20cl_L* | *caudolateral of area 20* | -59, -42, -16 |
| *A7ip_L* | *intraparietal area 7(hIP3)* | -27, -59, 54 |
| *A39c_L* | *caudal area 39(PGp)* | -34, -80, 29 |
| *A39rd_L* | *rostrodorsal area 39(Hip3)* | -38, -61, 46 |
| *A40c_L* | *caudal area 40(PFm)* | -56, -49, 38 |
| *A39rv_L* | *rostroventral area 39(PGa)* | -47, -65, 26 |

**Table S6. Nodes and their Montreal Neurological Institute (MNI) locations in the right executive network.**

| **Area** | **Anatomical and modified Cyto-architectonic descriptions** | Right hemisphere  X, Y, Z |
| --- | --- | --- |
| *A8dl_R* | *dorsolateral area 8* | 22, 26, 51 |
| *A9m_R* | *medial area 9* | 6, 38, 35 |
| *A9/46d_R* | *dorsal area 9/46* | 30, 37, 36 |
| *IFJ_R* | *inferior frontal junction* | 42, 11, 39 |
| *A46_R* | *area 46* | 28, 55, 17 |
| *A9/46v_R* | *ventral area 9/46* | 42, 44, 14 |
| *A8vl_R* | *ventrolateral area 8* | 42, 27, 39 |
| *A6vl_R* | *ventrolateral area 6* | 34, 8, 54 |
| *A10l_R* | *lateral area10* | 25, 61, -4 |
| *IFS_R* | *inferior frontal sulcus* | 48, 35, 13 |
| *A45r_R* | *rostral area 45* | 51, 36, -1 |
| *A12/47l_R* | *lateral area 12/47* | 42, 31, -9 |
| *A21c_R* | *caudal area 21* | 65, -29, -13 |
| *A37vl_R* | *ventrolateral area 37* | 54, -57, -8 |
| *A20cl_R* | *caudolateral of area 20* | 61, -40, -17 |
| *A7ip_R* | *intraparietal area 7(hIP3)* | 31, -54, 53 |
| *A39c_R* | *caudal area 39(PGp)* | 45, -71, 20 |
| *A39rd_R* | *rostrodorsal area 39(Hip3)* | 39, -65, 44 |
| *A40c_R* | *caudal area 40(PFm)* | 57, -44, 38 |
| *A39rv_R* | *rostroventral area 39(PGa)* | 53, -54, 25 |

**Table S7. Nodes and their Montreal Neurological Institute (MNI) locations in the visual network.**

| **Area** | **Anatomical and modified Cyto-architectonic descriptions** | **Left hemisphere** X, Y, Z | **Right hemisphere** X, Y, Z |
| --- | --- | --- | --- |
| *cLinG_L(R)* | *caudal lingual gyrus* | -11, -82, -11 | 10, -85, -9 |
| *rCunG_L(R)* | *rostral cuneus gyrus* | -5, -81, 10 | 7, -76, 11 |
| *cCunG_L(R)* | *caudal cuneus gyrus* | -6, -94, 1 | 8, -90, 12 |
| *rLinG_L(R)* | *rostral lingual gyrus* | -17, -60, -6 | 18, -60, -7 |
| *vmPOS_L(R)* | *ventromedial parietooccipital sulcus* | -13, -68, 12 | 15, -63, 12 |
| *mOccG_L(R)* | *middle occipital gyrus* | -31, -89, 11 | 34, -86, 11 |
| *V5/MT+_L(R)* | *area V5/MT+* | -46, -74, 3 | 48, -70, -1 |
| *OPC_L(R)* | *occipital polar cortex* | -18, -99, 2 | 22, -97, 4 |
| *iOccG_L(R)* | *inferior occipital gyrus* | -30, -88, -12 | 32, -85, -12 |
| *msOccG_L(R)* | *medial superior occipital gyrus* | -11, -88, 31 | 16, -85, 34 |
| *lsOccG_L(R)* | *lateral superior occipital gyrus* | -22, -77, 36 | 29, -75, 36 |

**Table S8. Comparison of global properties in default mode networks of patient and healthy control groups. (Glioma located in the right hemisphere)**

|  | **GE group** | **GnE group** | **HC group** | **One-way**  **ANOVA**  **(*P* value)**^†^ | **GE vs GnE**  **(*P*value)**^‡^ | **GE vs HC**  **(*P* value)**^‡^ | **GnE vs HC**  **(*P* value)**^‡^ |
| --- | --- | --- | --- | --- | --- | --- | --- |
| Global efficiency | 0.123±0.003 | 0.127±0.003 | 0.131±0.002 | 0.1282 | - | - | - |
| Local efficiency | 0.165±0.002 | 0.166±0.004 | 0.174±0.002 | 0.0355 | 0.6743 | 0.0143 | 0.0560 |
| Cp | 0.136±0.002 | 0.137±0.003 | 0.145±0.002 | 0.0048 | 0.9278 | 0.0031 | 0.0070 |
| Gamma | 0.367±0.014 | 0.369±0.02 | 0.393±0.01 | 0.3857 | - | - | - |
| Lambda | 0.26±0.003 | 0.258±0.002 | 0.262±0.003 | 0.6097 | - | - | - |
| Sigma | 0.338±0.013 | 0.343±0.018 | 0.361±0.011 | 0.4671 | - | - | - |
| Lp | 0.493±0.015 | 0.472±0.015 | 0.457±0.008 | 0.1391 | - | - | - |

Note. data are means±standard errors. The GE group = glioma-related epilepsy group. The GnE group = glioma with no related epilepsy. The HC group = healthy control group.

^†^ *p* < 0.05 (false-positive-adjustment corrected).

^‡^ *p* < 0.05 (least significant difference corrected)

**Table S9. Comparison of global properties in visual networks of patient and healthy control groups. (Glioma located in the right hemisphere)**

|  | **GE group** | **GnE group** | **HC group** | **One-way**  **ANOVA**  **(*P* value)**^†^ | **GE vs GnE**  **(*P* value)**^‡^ | **GE vs HC**  **(*P* value)**^‡^ | **GnE vs HC**  **(*P* value)**^‡^ |
| --- | --- | --- | --- | --- | --- | --- | --- |
| Global efficiency | 0.123±0.002 | 0.127±0.002 | 0.121±0.002 | 0.2233 | - | - | - |
| Local efficiency | 0.162±0.002 | 0.166±0.003 | 0.168±0.004 | 0.2702 | - | - | - |
| Cp | 0.138±0.002 | 0.14±0.003 | 0.146±0.004 | 0.1099 | - | - | - |
| Gamma | 0.402±0.02 | 0.439±0.025 | 0.454±0.027 | 0.2697 | - | - | - |
| Lambda | 0.263±0.004 | 0.267±0.005 | 0.287±0.007 | 0.0069 | 0.7041 | 0.0015 | 0.0007 |
| Sigma | 0.365±0.018 | 0.392±0.018 | 0.377±0.018 | 0.5985 | - | - | - |
| Lp | 0.49±0.01 | 0.475±0.01 | 0.507±0.012 | 0.1416 | - | - | - |

Note. data are means±standard errors. The GE group = glioma-related epilepsy group. The GnE group = glioma with no related epilepsy. The HC group = healthy control group.

^†^ *p* < 0.05 (false-positive-adjustment corrected).

^‡^ *p* < 0.05 (least significant difference corrected)

**Table S10. Comparison of global properties in visual networks of patient and healthy control groups. (Glioma located in the left hemisphere)**

|  | **GE group** | **GnE group** | **HC group** | **One-way**  **ANOVA**  **(*P* value)**^†^ | **GE vs GnE**  **(*P* value)**^‡^ | **GE vs HC**  **(*P* value)**^‡^ | **GnE vs HC**  **(*P* value)**^‡^ |
| --- | --- | --- | --- | --- | --- | --- | --- |
| Global efficiency | 0.126±0.002 | 0.128±0.003 | 0.121±0.002 | 0.1187 | - | - | - |
| Local efficiency | 0.161±0.003 | 0.165±0.003 | 0.168±0.004 | 0.3178 | - | - | - |
| Cp | 0.136±0.003 | 0.14±0.003 | 0.146±0.004 | 0.0815 | - | - | - |
| Gamma | 0.414±0.025 | 0.431±0.023 | 0.454±0.027 | 0.5318 | - | - | - |
| Lambda | 0.264±0.004 | 0.262±0.003 | 0.287±0.007 | 0.0009 | 0.7042 | 0.0015 | 0.0007 |
| Sigma | 0.375±0.022 | 0.393±0.019 | 0.377±0.018 | 0.7960 | - | - | - |
| Lp | 0.477±0.009 | 0.468±0.016 | 0.507±0.012 | 0.0821 | - | - | - |

Note. data are means±standard errors. The GE group = glioma-related epilepsy group. The GnE group = glioma with no related epilepsy. The HC group = healthy control group.

^†^ *p* < 0.05 (false-positive-adjustment corrected).

^‡^ *p* < 0.05 (least significant difference corrected)

**Table S11. Comparison of nodal efficiency in sensorimotor networks of patient and healthy groups. (Glioma located in the right hemisphere)**

| **Area** | **GE group** | **GnE group** | **Health group** | **One-way ANOVA (P value)**^†^ | **GE vs GnE (P value)**^‡^ | **GE vs Health (P value)**^‡^ | **GnE vs Health (P value)**^‡^ |
| --- | --- | --- | --- | --- | --- | --- | --- |
| *A6m_L* | 0.147±0.002 | 0.142±0.005 | 0.149±0.002 | 0.3459 | - | - | - |
| *A4hf_L* | 0.137±0.003 | 0.133±0.005 | 0.139±0.003 | 0.3863 | - | - | - |
| *A6cdl_L* | 0.137±0.005 | 0.137±0.005 | 0.13±0.005 | 0.5351 | - | - | - |
| *A4ul_L* | 0.143±0.004 | 0.143±0.003 | 0.14±0.004 | 0.8511 | - | - | - |
| *A4t_L* | 0.138±0.004 | 0.119±0.012 | 0.134±0.006 | 0.1872 | - | - | - |
| *A4tl_L* | 0.112±0.006 | 0.128±0.004 | 0.128±0.006 | 0.0627 | - | - | - |
| *A6cvl_L* | 0.072±0.009 | 0.113±0.005 | 0.061±0.01 | 0.0008 | 0.0029 | 0.3886 | 0.0003 |
| *A1/2/3ll_L* | 0.137±0.003 | 0.118±0.008 | 0.137±0.003 | 0.0191 | - | - | - |
| *A4ll_L* | 0.136±0.004 | 0.143±0.003 | 0.143±0.002 | 0.1448 | - | - | - |
| *A1/2/3ulh_Lf* | 0.133±0.003 | 0.142±0.003 | 0.131±0.002 | 0.0366 | - | - | - |
| *A1/2/3tonIa_L* | 0.129±0.004 | 0.137±0.005 | 0.12±0.005 | 0.0606 | - | - | - |
| *A2_L* | 0.122±0.006 | 0.132±0.007 | 0.119±0.005 | 0.3257 | - | - | - |
| *A1/2/3t_Lru* | 0.146±0.002 | 0.147±0.005 | 0.149±0.003 | 0.8112 | - | - | - |
| *A23d_L* | 0.028±0.008 | 0.034±0.01 | 0.035±0.01 | 0.8186 | - | - | - |
| *A23v_L* | 0.026±0.007 | 0.041±0.012 | 0.055±0.012 | 0.1345 | - | - | - |
| *A24cd_L* | 0.119±0.006 | 0.114±0.009 | 0.12±0.006 | 0.774 | - | - | - |
| *A23c_L* | 0.1±0.009 | 0.089±0.011 | 0.109±0.01 | 0.3912 | - | - | - |
| *A32sg_L* | 0.034±0.006 | 0.04±0.011 | 0.034±0.011 | 0.8688 | - | - | - |
| *mPMtha_L* | 0.008±0.004 | 0.007±0.005 | 0.004±0.002 | 0.8085 | - | - | - |
| *Stha_L* | 0.005±0.003 | 0.011±0.007 | 0.011±0.005 | 0.673 | - | - | - |

Note. data are means±standard errors. The GE group = glioma-related epilepsy group. The GnE group = glioma with no related epilepsy. The HC group = healthy control group.

^†^ *p* < 0.05 (false-positive-adjustment corrected).

^‡^ *p* < 0.05 (least significant difference corrected)

**Table S12. Comparison of nodal local efficiency in sensorimotor networks of patient and healthy groups. (Glioma located in the right hemisphere)**

| **Area** | **GE group** | **GnE group** | **Health group** | **One-way ANOVA (P value)**^†^ | **GE vs GnE (P value)**^‡^ | **GE vs Health (P value)**^‡^ | **GnE vs Health (P value)**^‡^ |
| --- | --- | --- | --- | --- | --- | --- | --- |
| *A6m_L* | 0.197±0.003 | 0.193±0.004 | 0.187±0.004 | 0.1662 | - | - | - |
| *A4hf_L* | 0.198±0.004 | 0.193±0.009 | 0.199±0.006 | 0.747 | - | - | - |
| *A6cdl_L* | 0.191±0.008 | 0.202±0.007 | 0.179±0.013 | 0.2762 | - | - | - |
| *A4ul_L* | 0.203±0.005 | 0.211±0.004 | 0.197±0.006 | 0.187 | - | - | - |
| *A4t_L* | 0.208±0.006 | 0.197±0.019 | 0.203±0.014 | 0.8434 | - | - | - |
| *A4tl_L* | 0.169±0.009 | 0.183±0.011 | 0.191±0.01 | 0.2964 | - | - | - |
| *A6cvl_L* | 0.082±0.019 | 0.187±0.013 | 0.083±0.02 | 0.0003 | 0.0002 | 0.9739 | 0.0003 |
| *A1/2/3ll_L* | 0.2±0.007 | 0.16±0.02 | 0.202±0.008 | 0.0335 | - | - | - |
| *A4ll_L* | 0.207±0.006 | 0.205±0.005 | 0.213±0.004 | 0.5774 | - | - | - |
| *A1/2/3ulh_Lf* | 0.204±0.004 | 0.203±0.006 | 0.201±0.008 | 0.9234 | - | - | - |
| *A1/2/3tonIa_L* | 0.195±0.007 | 0.201±0.006 | 0.173±0.016 | 0.1715 | - | - | - |
| *A2_L* | 0.201±0.014 | 0.197±0.01 | 0.2±0.015 | 0.9759 | - | - | - |
| *A1/2/3t_Lru* | 0.205±0.003 | 0.201±0.006 | 0.205±0.004 | 0.7965 | - | - | - |
| *A23d_L* | 0.032±0.015 | 0.049±0.022 | 0.051±0.02 | 0.725 | - | - | - |
| *A23v_L* | 0.028±0.012 | 0.054±0.021 | 0.07±0.018 | 0.1854 | - | - | - |
| *A24cd_L* | 0.192±0.008 | 0.152±0.019 | 0.188±0.01 | 0.0582 | - | - | - |
| *A23c_L* | 0.148±0.019 | 0.1±0.018 | 0.137±0.018 | 0.1946 | - | - | - |
| *A32sg_L* | 0.041±0.014 | 0.063±0.022 | 0.051±0.019 | 0.7143 | - | - | - |
| *mPMtha_L* | 0.006±0.005 | 0.006±0.006 | 0±0 | 0.4549 | - | - | - |
| *Stha_L* | 0.004±0.003 | 0.005±0.005 | 0.007±0.006 | 0.8446 | - | - | - |

Note. data are means±standard errors. The GE group = glioma-related epilepsy group. The GnE group = glioma with no related epilepsy. The HC group = healthy control group.

^†^ *p* < 0.05 (false-positive-adjustment corrected).

^‡^ *p* < 0.05 (least significant difference corrected)

**Table S13. Comparison of nodal clustering coefficient in sensorimotor networks of patient and healthy groups. (Glioma located in the right hemisphere)**

| **Area** | **GE group** | **GnE group** | **Health group** | **One-way ANOVA (P value)**^†^ | **GE vs GnE (P value)**^‡^ | **GE vs Health (P value)**^‡^ | **GnE vs Health (P value)**^‡^ |
| --- | --- | --- | --- | --- | --- | --- | --- |
| *A6m_L* | 0.161±0.005 | 0.16±0.006 | 0.151±0.005 | 0.3268 | - | - | - |
| *A4hf_L* | 0.169±0.006 | 0.17±0.01 | 0.168±0.008 | 0.98 | - | - | - |
| *A6cdl_L* | 0.164±0.008 | 0.177±0.008 | 0.156±0.012 | 0.3295 | - | - | - |
| *A4ul_L* | 0.172±0.006 | 0.185±0.007 | 0.171±0.008 | 0.3328 | - | - | - |
| *A4t_L* | 0.185±0.009 | 0.183±0.018 | 0.181±0.013 | 0.973 | - | - | - |
| *A4tl_L* | 0.15±0.007 | 0.159±0.011 | 0.165±0.011 | 0.5333 | - | - | - |
| *A6cvl_L* | 0.076±0.017 | 0.175±0.012 | 0.08±0.019 | 0.0003 | 0.0002 | 0.8729 | 0.0004 |
| *A1/2/3ll_L* | 0.174±0.009 | 0.145±0.019 | 0.181±0.01 | 0.1234 | - | - | - |
| *A4ll_L* | 0.184±0.009 | 0.176±0.009 | 0.189±0.007 | 0.5786 | - | - | - |
| *A1/2/3ulh_Lf* | 0.179±0.005 | 0.176±0.008 | 0.178±0.008 | 0.9569 | - | - | - |
| *A1/2/3tonIa_L* | 0.17±0.009 | 0.177±0.008 | 0.151±0.014 | 0.2328 | - | - | - |
| *A2_L* | 0.185±0.013 | 0.175±0.012 | 0.186±0.014 | 0.8198 | - | - | - |
| *A1/2/3t_Lru* | 0.173±0.005 | 0.171±0.009 | 0.172±0.007 | 0.9676 | - | - | - |
| *A23d_L* | 0.031±0.014 | 0.047±0.021 | 0.049±0.02 | 0.7296 | - | - | - |
| *A23v_L* | 0.028±0.012 | 0.048±0.019 | 0.065±0.017 | 0.2192 | - | - | - |
| *A24cd_L* | 0.17±0.009 | 0.131±0.017 | 0.17±0.01 | 0.0379 | - | - | - |
| *A23c_L* | 0.137±0.018 | 0.089±0.016 | 0.12±0.015 | 0.1372 | - | - | - |
| *A32sg_L* | 0.04±0.014 | 0.062±0.022 | 0.048±0.018 | 0.7033 | - | - | - |
| *mPMtha_L* | 0.006±0.005 | 0.006±0.006 | 0±0 | 0.4549 | - | - | - |
| *Stha_L* | 0.004±0.003 | 0.004±0.004 | 0.007±0.006 | 0.8237 | - | - | - |

Note. data are means±standard errors. The GE group = glioma-related epilepsy group. The GnE group = glioma with no related epilepsy. The HC group = healthy control group.

^†^ *p* < 0.05 (false-positive-adjustment corrected).

^‡^ *p* < 0.05 (least significant difference corrected)

**Table S14. Comparison of betweenness centrality in default mode networks of patient and healthy groups. (Glioma located in the right hemisphere)**

| **Area** | **GE group** | **GnE group** | **Health group** | **One-way ANOVA (P value)**^†^ | **GE vs GnE (P value)**^‡^ | **GE vs Health (P value)**^‡^ | **GnE vs Health (P value)**^‡^ |
| --- | --- | --- | --- | --- | --- | --- | --- |
| *A8m_L* | 3.425±0.62 | 4.163±0.898 | 7.052±0.957 | 0.0067 | 0.5394 | 0.0025 | 0.0213 |
| *A8dl_L* | 3.051±0.79 | 4.248±1.422 | 3.294±1.143 | 0.7382 | - | - | - |
| *A9l_L* | 1.742±0.432 | 2.044±0.582 | 0.911±0.258 | 0.1685 | - | - | - |
| *A6dl_L* | 3.415±0.768 | 3.286±0.67 | 4.816±1.12 | 0.4139 | - | - | - |
| *A6m_L* | 2.22±0.869 | 1.775±0.548 | 2.289±0.501 | 0.8616 | - | - | - |
| *A9m_L* | 4.299±0.692 | 3.747±0.726 | 5.495±1.223 | 0.4168 | - | - | - |
| *A10m_L* | 2.091±0.425 | 3.007±1.035 | 2.404±0.727 | 0.6778 | - | - | - |
| *A9/46d_L* | 3.848±0.7 | 2.931±0.644 | 1.895±0.5 | 0.0844 | - | - | - |
| *A9/46v_L* | 1.754±0.332 | 1.662±0.329 | 1.533±0.314 | 0.8843 | - | - | - |
| *A8vl_L* | 2.653±0.425 | 4.561±0.888 | 4.873±0.774 | 0.0505 | - | - | - |
| *A6vl_L* | 3.975±0.709 | 4.245±0.72 | 4.92±0.863 | 0.662 | - | - | - |
| *A20iv_L* | 2.392±0.724 | 2.934±1.248 | 2.314±0.566 | 0.8627 | - | - | - |
| *A37elv_L* | 4.77±0.871 | 4.613±1.092 | 3.002±0.707 | 0.2902 | - | - | - |
| *A20r_L* | 3.297±0.823 | 2.173±0.768 | 2.311±0.736 | 0.537 | - | - | - |
| *A20il_L* | 3.446±1.037 | 3.033±0.69 | 3.622±1.19 | 0.9248 | - | - | - |
| *A37vl_L* | 2.083±0.529 | 1.437±0.384 | 2.349±0.695 | 0.5431 | - | - | - |
| *A20cl_L* | 2.476±0.594 | 2.971±0.607 | 4.278±1.303 | 0.3492 | - | - | - |
| *A20cv_L* | 3.026±0.659 | 5.256±1.115 | 5.266±0.837 | 0.1028 | - | - | - |
| *A35/36r_L* | 1.784±0.624 | 0.892±0.435 | 2.774±1.059 | 0.2668 | - | - | - |
| *A35/36c_L* | 0.906±0.303 | 4.361±1.206 | 5.593±1.07 | 0.0012 | - | - | - |
| *TL_L* | 1.585±0.529 | 2.052±0.621 | 4.576±1.284 | 0.0447 | - | - | - |
| *A28/34_L* | 2.366±0.684 | 1.912±0.645 | 2.933±0.951 | 0.6731 | - | - | - |
| *TI_L* | 0.831±0.293 | 0.577±0.29 | 0.859±0.368 | 0.8104 | - | - | - |
| *TH_L* | 1.205±0.395 | 1.875±0.693 | 1.95±0.851 | 0.6702 | - | - | - |
| *A5l_L* | 0.963±0.243 | 2.173±0.947 | 1.038±0.285 | 0.2177 | - | - | - |
| *A23d_L* | 1.512±0.594 | 1.154±0.309 | 2.056±0.583 | 0.5143 | - | - | - |
| *A24rv_L* | 1.709±0.603 | 1.839±0.497 | 1.445±0.36 | 0.8585 | - | - | - |
| *A24cd_L* | 3.02±1.006 | 3.653±0.761 | 2.402±0.777 | 0.6253 | - | - | - |
| *A23c_L* | 4.021±0.666 | 6.245±1.467 | 6.184±0.882 | 0.1911 | - | - | - |

Note. data are means±standard errors. The GE group = glioma-related epilepsy group. The GnE group = glioma with no related epilepsy. The HC group = healthy control group.

^†^ *p* < 0.05 (false-positive-adjustment corrected).

^‡^ *p* < 0.05 (least significant difference corrected)

**Table S15. Comparison of degree centrality in visual networks of patient and healthy groups. (Glioma located in the left hemisphere)**

| **Area** | **GE group** | **GnE group** | **Health group** | **One-way ANOVA (P value)**^†^ | **GE vs GnE (P value)**^‡^ | **GE vs Health (P value)**^‡^ | **GnE vs Health (P value)**^‡^ |
| --- | --- | --- | --- | --- | --- | --- | --- |
| *cLinG_L* | 1.375±0.16 | 1.596±0.164 | 1.585±0.138 | 0.5161 | - | - | - |
| *cLinG_R* | 1.533±0.144 | 1.436±0.109 | 1.578±0.124 | 0.7367 | - | - | - |
| *rCunG_L* | 2.162±0.106 | 2.173±0.134 | 2.225±0.07 | 0.9012 | - | - | - |
| *rCunG_R* | 2.026±0.073 | 2.047±0.111 | 2.098±0.071 | 0.8267 | - | - | - |
| *cCunG_L* | 1.836±0.16 | 1.794±0.127 | 1.7±0.171 | 0.8156 | - | - | - |
| *cCunG_R* | 1.739±0.113 | 1.886±0.117 | 1.995±0.104 | 0.2648 | - | - | - |
| *rLinG_L* | 0.893±0.139 | 1.397±0.144 | 1.882±0.108 | <0.0001 | 0.0095 | <0.0001 | 0.0135 |
| *rLinG_R* | 1.262±0.191 | 1.573±0.16 | 1.643±0.138 | 0.2271 | - | - | - |
| *vmPOS_L* | 1.261±0.153 | 1.259±0.141 | 1.622±0.116 | 0.1142 | - | - | - |
| *vmPOS_R* | 1.292±0.12 | 1.339±0.112 | 1.536±0.117 | 0.2971 | - | - | - |
| *mOccG_L* | 1.534±0.128 | 1.582±0.143 | 1.324±0.135 | 0.3702 | - | - | - |
| *mOccG_R* | 1.436±0.107 | 1.302±0.156 | 1.317±0.171 | 0.7701 | - | - | - |
| *V5/MT+_L* | 0.933±0.14 | 0.687±0.146 | 0.855±0.164 | 0.5075 | - | - | - |
| *V5/MT+_R* | 1.109±0.153 | 0.825±0.168 | 0.71±0.152 | 0.1833 | - | - | - |
| *OPC_L* | 1.588±0.127 | 1.412±0.176 | 1.376±0.096 | 0.4855 | - | - | - |
| *OPC_R* | 1.717±0.117 | 1.524±0.146 | 1.437±0.145 | 0.3215 | - | - | - |
| *iOccG_L* | 1.63±0.195 | 1.236±0.142 | 1.071±0.148 | 0.0547 | - | - | - |
| *iOccG_R* | 1.324±0.146 | 1.319±0.164 | 1.206±0.098 | 0.792 | - | - | - |
| *msOccG_L* | 1.5±0.192 | 1.599±0.149 | 1.566±0.16 | 0.9151 | - | - | - |
| *msOccG_R* | 1.523±0.156 | 1.644±0.17 | 1.431±0.179 | 0.6796 | - | - | - |
| *lsOccG_L* | 0.814±0.155 | 0.849±0.171 | 0.678±0.099 | 0.6803 | - | - | - |
| *lsOccG_R* | 0.834±0.154 | 0.838±0.15 | 0.49±0.08 | 0.1153 | - | - | - |

Note. data are means±standard errors. The GE group = glioma-related epilepsy group. The GnE group = glioma with no related epilepsy. The HC group = healthy control group.

^†^ *p* < 0.05 (false-positive-adjustment corrected).

^‡^ *p* < 0.05 (least significant difference corrected)

**Table S16. Comparison of nodal efficiency in visual networks of patient and healthy groups. (Glioma located in the left hemisphere)**

| **Area** | **GE group** | **GnE group** | **Health group** | **One-way ANOVA (P value)**^†^ | **GE vs GnE (P value)**^‡^ | **GE vs Health (P value)**^‡^ | **GnE vs Health (P value)**^‡^ |
| --- | --- | --- | --- | --- | --- | --- | --- |
| *cLinG_L* | 0.128±0.009 | 0.138±0.005 | 0.133±0.006 | 0.5823 |  |  |  |
| *cLinG_R* | 0.136±0.004 | 0.134±0.003 | 0.134±0.003 | 0.8802 |  |  |  |
| *rCunG_L* | 0.153±0.003 | 0.156±0.004 | 0.152±0.003 | 0.6978 |  |  |  |
| *rCunG_R* | 0.149±0.002 | 0.151±0.003 | 0.147±0.002 | 0.6239 |  |  |  |
| *cCunG_L* | 0.144±0.006 | 0.146±0.004 | 0.131±0.01 | 0.2948 |  |  |  |
| *cCunG_R* | 0.143±0.003 | 0.149±0.003 | 0.147±0.003 | 0.4297 |  |  |  |
| *rLinG_L* | 0.101±0.009 | 0.129±0.006 | 0.14±0.005 | 0.0005 | 0.0052 | 0.0002 | 0.2813 |
| *rLinG_R* | 0.114±0.011 | 0.135±0.006 | 0.132±0.005 | 0.1503 |  |  |  |
| *vmPOS_L* | 0.122±0.006 | 0.124±0.007 | 0.132±0.005 | 0.4759 |  |  |  |
| *vmPOS_R* | 0.126±0.005 | 0.127±0.005 | 0.128±0.005 | 0.9554 |  |  |  |
| *mOccG_L* | 0.134±0.004 | 0.136±0.008 | 0.119±0.007 | 0.1484 |  |  |  |
| *mOccG_R* | 0.129±0.005 | 0.126±0.007 | 0.118±0.008 | 0.4915 |  |  |  |
| *V5/MT+_L* | 0.106±0.007 | 0.089±0.011 | 0.085±0.013 | 0.2763 |  |  |  |
| *V5/MT+_R* | 0.112±0.008 | 0.092±0.013 | 0.083±0.011 | 0.1432 |  |  |  |
| *OPC_L* | 0.138±0.003 | 0.132±0.006 | 0.124±0.005 | 0.117 |  |  |  |
| *OPC_R* | 0.141±0.004 | 0.137±0.005 | 0.126±0.005 | 0.0949 |  |  |  |
| *iOccG_L* | 0.134±0.008 | 0.122±0.01 | 0.105±0.009 | 0.0753 |  |  |  |
| *iOccG_R* | 0.129±0.005 | 0.123±0.011 | 0.118±0.005 | 0.5362 |  |  |  |
| *msOccG_L* | 0.127±0.011 | 0.139±0.005 | 0.129±0.006 | 0.5491 |  |  |  |
| *msOccG_R* | 0.129±0.01 | 0.139±0.006 | 0.122±0.008 | 0.3603 |  |  |  |
| *lsOccG_L* | 0.089±0.013 | 0.095±0.013 | 0.085±0.008 | 0.8139 |  |  |  |
| *lsOccG_R* | 0.092±0.011 | 0.102±0.01 | 0.068±0.009 | 0.0575 |  |  |  |

Note. data are means±standard errors. The GE group = glioma-related epilepsy group. The GnE group = glioma with no related epilepsy. The HC group = healthy control group.

^†^ *p* < 0.05 (false-positive-adjustment corrected).

^‡^ *p* < 0.05 (least significant difference corrected)

**Table S17. Comparison of nodal local efficiency in visual networks of patient and healthy groups. (Glioma located in the left hemisphere)**

| **Area** | **GE group** | **GnE group** | **Health group** | **One-way ANOVA (P value)**^†^ | **GE vs GnE (P value)**^‡^ | **GE vs Health (P value)**^‡^ | **GnE vs Health (P value)**^‡^ |
| --- | --- | --- | --- | --- | --- | --- | --- |
| *cLinG_L* | 0.163±0.014 | 0.2±0.006 | 0.166±0.013 | 0.0623 | - | - | - |
| *cLinG_R* | 0.165±0.012 | 0.191±0.01 | 0.188±0.008 | 0.1692 | - | - | - |
| *rCunG_L* | 0.186±0.006 | 0.181±0.006 | 0.196±0.004 | 0.1449 | - | - | - |
| *rCunG_R* | 0.185±0.007 | 0.191±0.005 | 0.202±0.003 | 0.0755 | - | - | - |
| *cCunG_L* | 0.172±0.014 | 0.187±0.008 | 0.158±0.014 | 0.2873 | - | - | - |
| *cCunG_R* | 0.202±0.006 | 0.185±0.007 | 0.196±0.007 | 0.1786 | - | - | - |
| *rLinG_L* | 0.141±0.017 | 0.187±0.016 | 0.199±0.008 | 0.0124 | - | - | - |
| *rLinG_R* | 0.14±0.017 | 0.19±0.008 | 0.2±0.007 | 0.0012 | 0.0040 | 0.0005 | 0.5628 |
| *vmPOS_L* | 0.194±0.01 | 0.183±0.017 | 0.216±0.005 | 0.1558 | - | - | - |
| *vmPOS_R* | 0.196±0.009 | 0.194±0.013 | 0.211±0.007 | 0.4385 | - | - | - |
| *mOccG_L* | 0.158±0.013 | 0.163±0.01 | 0.151±0.015 | 0.8135 | - | - | - |
| *mOccG_R* | 0.167±0.012 | 0.16±0.01 | 0.154±0.014 | 0.7567 | - | - | - |
| *V5/MT+_L* | 0.143±0.018 | 0.106±0.022 | 0.102±0.024 | 0.3202 | - | - | - |
| *V5/MT+_R* | 0.137±0.018 | 0.106±0.021 | 0.104±0.023 | 0.4312 | - | - | - |
| *OPC_L* | 0.173±0.012 | 0.174±0.013 | 0.191±0.009 | 0.4732 | - | - | - |
| *OPC_R* | 0.176±0.008 | 0.186±0.008 | 0.179±0.009 | 0.7181 | - | - | - |
| *iOccG_L* | 0.157±0.014 | 0.153±0.018 | 0.173±0.02 | 0.6919 | - | - | - |
| *iOccG_R* | 0.171±0.011 | 0.153±0.017 | 0.168±0.014 | 0.6424 | - | - | - |
| *msOccG_L* | 0.167±0.014 | 0.163±0.013 | 0.184±0.014 | 0.5518 | - | - | - |
| *msOccG_R* | 0.159±0.017 | 0.161±0.015 | 0.16±0.018 | 0.9944 | - | - | - |
| *lsOccG_L* | 0.093±0.02 | 0.092±0.017 | 0.106±0.021 | 0.8548 | - | - | - |
| *lsOccG_R* | 0.099±0.019 | 0.115±0.02 | 0.099±0.023 | 0.824 | - | - | - |

Note. data are means±standard errors. The GE group = glioma-related epilepsy group. The GnE group = glioma with no related epilepsy. The HC group = healthy control group.

^†^ *p* < 0.05 (false-positive-adjustment corrected).

^‡^ *p* < 0.05 (least significant difference corrected)

**Table S18. Comparison of nodal clustering coefficient in visual networks of patient and healthy groups. (Glioma located in the left hemisphere)**

| **Area** | **GE group** | **GnE group** | **Health group** | **One-way ANOVA (P value)**^†^ | **GE vs GnE (P value)**^‡^ | **GE vs Health (P value)**^‡^ | **GnE vs Health (P value)**^‡^ |
| --- | --- | --- | --- | --- | --- | --- | --- |
| cLinG_L | 0.137±0.013 | 0.174±0.008 | 0.137±0.012 | 0.0421 | - | - | - |
| cLinG_R | 0.138±0.012 | 0.168±0.012 | 0.157±0.009 | 0.1633 | - | - | - |
| rCunG_L | 0.146±0.007 | 0.139±0.009 | 0.158±0.006 | 0.1912 | - | - | - |
| rCunG_R | 0.147±0.008 | 0.15±0.007 | 0.167±0.005 | 0.1033 | - | - | - |
| cCunG_L | 0.14±0.013 | 0.152±0.01 | 0.13±0.012 | 0.445 | - | - | - |
| cCunG_R | 0.173±0.008 | 0.149±0.008 | 0.164±0.008 | 0.1164 | - | - | - |
| rLinG_L | 0.129±0.015 | 0.168±0.016 | 0.173±0.01 | 0.0547 | - | - | - |
| rLinG_R | 0.117±0.014 | 0.166±0.01 | 0.178±0.008 | 0.0007 | 0.0037 | 0.0003 | 0.4519 |
| vmPOS_L | 0.175±0.011 | 0.166±0.017 | 0.196±0.009 | 0.2412 | - | - | - |
| vmPOS_R | 0.179±0.01 | 0.175±0.013 | 0.194±0.008 | 0.436 | - | - | - |
| mOccG_L | 0.128±0.011 | 0.128±0.01 | 0.124±0.013 | 0.9577 | - | - | - |
| mOccG_R | 0.137±0.011 | 0.132±0.009 | 0.132±0.014 | 0.9316 | - | - | - |
| V5/MT+_L | 0.129±0.017 | 0.098±0.021 | 0.09±0.021 | 0.3223 | - | - | - |
| V5/MT+_R | 0.119±0.017 | 0.094±0.019 | 0.096±0.021 | 0.5795 | - | - | - |
| OPC_L | 0.144±0.011 | 0.149±0.013 | 0.165±0.01 | 0.4063 | - | - | - |
| OPC_R | 0.142±0.009 | 0.156±0.01 | 0.151±0.009 | 0.54 | - | - | - |
| iOccG_L | 0.125±0.013 | 0.13±0.017 | 0.159±0.02 | 0.2971 | - | - | - |
| iOccG_R | 0.148±0.012 | 0.132±0.016 | 0.15±0.014 | 0.6309 | - | - | - |
| msOccG_L | 0.141±0.013 | 0.134±0.012 | 0.164±0.015 | 0.2673 | - | - | - |
| msOccG_R | 0.13±0.015 | 0.135±0.015 | 0.141±0.016 | 0.8845 | - | - | - |
| lsOccG_L | 0.078±0.017 | 0.075±0.014 | 0.099±0.02 | 0.557 | - | - | - |
| lsOccG_R | 0.085±0.016 | 0.102±0.017 | 0.091±0.022 | 0.7983 | - | - | - |

Note. data are means±standard errors. The GE group = glioma-related epilepsy group. The GnE group = glioma with no related epilepsy. The HC group = healthy control group.

^†^ *p* < 0.05 (false-positive-adjustment corrected).

^‡^ *p* < 0.05 (least significant difference corrected)

**Table S19. Comparison of nodal clustering coefficient in default mode networks of patient and healthy groups. (Glioma located in the left hemisphere)**

| **Area** | **GE group** | **GnE group** | **Health group** | **One-way ANOVA (P value)**^†^ | **GE vs GnE (P value)**^‡^ | **GE vs Health (P value)**^‡^ | **GnE vs Health (P value)**^‡^ |
| --- | --- | --- | --- | --- | --- | --- | --- |
| A8m_R | 0.16±0.005 | 0.148±0.007 | 0.127±0.006 | 0.0013 | 0.1562 | 0.0003 | 0.0225 |
| A8dl_R | 0.156±0.006 | 0.158±0.006 | 0.162±0.008 | 0.7932 | - | - | - |
| A9l_R | 0.192±0.007 | 0.168±0.007 | 0.166±0.008 | 0.0159 | - | - | - |
| A6dl_R | 0.147±0.01 | 0.164±0.008 | 0.155±0.008 | 0.3769 | - | - | - |
| A6m_R | 0.168±0.009 | 0.169±0.014 | 0.169±0.009 | 0.9992 | - | - | - |
| A9m_R | 0.16±0.008 | 0.152±0.006 | 0.143±0.007 | 0.2945 | - | - | - |
| A10m_R | 0.184±0.008 | 0.167±0.009 | 0.182±0.008 | 0.2854 | - | - | - |
| A9/46d_R | 0.152±0.007 | 0.152±0.008 | 0.149±0.007 | 0.9301 | - | - | - |
| A9/46v_R | 0.168±0.009 | 0.16±0.006 | 0.166±0.009 | 0.7702 | - | - | - |
| A8vl_R | 0.165±0.007 | 0.16±0.008 | 0.163±0.007 | 0.8984 | - | - | - |
| A6vl_R | 0.151±0.006 | 0.131±0.005 | 0.145±0.006 | 0.0359 | - | - | - |
| A20iv_R | 0.104±0.017 | 0.102±0.019 | 0.078±0.017 | 0.5141 | - | - | - |
| A37elv_R | 0.137±0.01 | 0.141±0.013 | 0.132±0.01 | 0.8523 | - | - | - |
| A20r_R | 0.112±0.012 | 0.097±0.015 | 0.126±0.014 | 0.3264 | - | - | - |
| A20il_R | 0.15±0.008 | 0.156±0.008 | 0.137±0.012 | 0.3979 | - | - | - |
| A37vl_R | 0.139±0.01 | 0.178±0.01 | 0.144±0.016 | 0.0693 | - | - | - |
| A20cl_R | 0.166±0.008 | 0.15±0.006 | 0.149±0.008 | 0.1923 | - | - | - |
| A20cv_R | 0.163±0.008 | 0.14±0.009 | 0.139±0.011 | 0.14 | - | - | - |
| A35/36r_R | 0.076±0.012 | 0.099±0.019 | 0.096±0.018 | 0.5638 | - | - | - |
| A35/36c_R | 0.097±0.014 | 0.103±0.014 | 0.124±0.011 | 0.3041 | - | - | - |
| TL_R | 0.087±0.018 | 0.109±0.019 | 0.116±0.015 | 0.4363 | - | - | - |
| A28/34_R | 0.095±0.012 | 0.104±0.02 | 0.107±0.01 | 0.8169 | - | - | - |
| TI_R | 0.095±0.021 | 0.108±0.023 | 0.131±0.015 | 0.4189 | - | - | - |
| TH_R | 0.074±0.016 | 0.097±0.022 | 0.081±0.023 | 0.7114 | - | - | - |
| A5l_R | 0.163±0.01 | 0.168±0.011 | 0.145±0.018 | 0.448 | - | - | - |
| A23d_R | 0.171±0.007 | 0.162±0.009 | 0.165±0.012 | 0.7904 | - | - | - |
| A24rv_R | 0.144±0.014 | 0.15±0.013 | 0.122±0.018 | 0.397 | - | - | - |
| A24cd_R | 0.165±0.01 | 0.148±0.008 | 0.168±0.009 | 0.27 | - | - | - |
| A23c_R | 0.137±0.008 | 0.124±0.005 | 0.123±0.009 | 0.3365 | - | - | - |

Note. data are means±standard errors. The GE group = glioma-related epilepsy group. The GnE group = glioma with no related epilepsy. The HC group = healthy control group.

^†^ *p* < 0.05 (false-positive-adjustment corrected).

^‡^ *p* < 0.05 (least significant difference corrected)

**Table S20. Comparison of betweenness centrality in default mode networks of patient and healthy groups. (Glioma located in the left hemisphere)**

| **Area** | **GE group** | **GnE group** | **Health group** | **One-way ANOVA (P value)**^†^ | **GE vs GnE (P value)**^‡^ | **GE vs Health (P value)**^‡^ | **GnE vs Health (P value)**^‡^ |
| --- | --- | --- | --- | --- | --- | --- | --- |
| A8m_R | 3.093±0.561 | 3.712±0.713 | 7.133±0.899 | 0.0005 | 0.5545 | 0.0003 | 0.0022 |
| A8dl_R | 4.262±0.788 | 3.144±0.697 | 2.918±0.647 | 0.3622 | - | - | - |
| A9l_R | 1.399±0.367 | 1.892±0.556 | 2.409±0.646 | 0.3994 | - | - | - |
| A6dl_R | 4.657±0.925 | 2.29±0.599 | 3.498±0.84 | 0.1338 | - | - | - |
| A6m_R | 2.119±0.48 | 2.271±1.016 | 2.723±0.692 | 0.833 | - | - | - |
| A9m_R | 3.621±0.615 | 3.463±0.536 | 5±0.845 | 0.228 | - | - | - |
| A10m_R | 2.422±0.765 | 2.837±0.906 | 1.36±0.337 | 0.3282 | - | - | - |
| A9/46d_R | 4.026±0.838 | 3.976±1.001 | 4.35±0.886 | 0.9513 | - | - | - |
| A9/46v_R | 2.077±0.452 | 1.775±0.548 | 1.662±0.417 | 0.809 | - | - | - |
| A8vl_R | 3.285±0.54 | 2.569±0.482 | 2.354±0.431 | 0.3671 | - | - | - |
| A6vl_R | 4.425±0.847 | 5.59±0.715 | 4.586±0.78 | 0.5415 | - | - | - |
| A20iv_R | 2.162±0.883 | 1.125±0.505 | 0.321±0.188 | 0.1108 | - | - | - |
| A37elv_R | 5.455±1.152 | 3.875±0.887 | 4.009±0.965 | 0.4755 | - | - | - |
| A20r_R | 2.709±0.752 | 2.69±0.849 | 2.89±0.774 | 0.9807 | - | - | - |
| A20il_R | 4.948±1.07 | 3.647±0.692 | 3.802±0.919 | 0.55 | - | - | - |
| A37vl_R | 3.359±0.856 | 1.145±0.301 | 1.847±0.482 | 0.0419 | - | - | - |
| A20cl_R | 2.874±0.637 | 2.168±0.38 | 3.596±1.073 | 0.4331 | - | - | - |
| A20cv_R | 4.239±1.138 | 3.955±0.914 | 4.2±0.81 | 0.9762 | - | - | - |
| A35/36r_R | 3.42±0.768 | 2.259±0.756 | 2.895±1.006 | 0.6333 | - | - | - |
| A35/36c_R | 2.99±0.69 | 3.418±0.881 | 2.858±0.707 | 0.8661 | - | - | - |
| TL_R | 2.475±0.769 | 2.235±0.6 | 3.447±0.752 | 0.4621 | - | - | - |
| A28/34_R | 2.764±0.732 | 2.372±0.794 | 3.823±1.022 | 0.4768 | - | - | - |
| TI_R | 1.06±0.385 | 1.274±0.638 | 1.615±0.441 | 0.717 | - | - | - |
| TH_R | 2.245±0.685 | 0.659±0.218 | 1.137±0.592 | 0.1253 | - | - | - |
| A5l_R | 1.798±0.657 | 1.869±0.443 | 2.263±0.755 | 0.857 | - | - | - |
| A23d_R | 2.913±0.878 | 2.26±0.471 | 1.147±0.37 | 0.1427 | - | - | - |
| A24rv_R | 1.591±0.783 | 2.445±0.689 | 1.288±0.431 | 0.4545 | - | - | - |
| A24cd_R | 1.948±0.431 | 4.917±1.041 | 2.279±0.735 | 0.016 | - | - | - |
| A23c_R | 6.29±1.05 | 7.084±1.259 | 6.109±1.179 | 0.8252 | - | - | - |

Note. data are means±standard errors. The GE group = glioma-related epilepsy group. The GnE group = glioma with no related epilepsy. The HC group = healthy control group.

^†^ *p* < 0.05 (false-positive-adjustment corrected).

^‡^ *p* < 0.05 (least significant difference corrected)
